# A method for identifying the cause of inefficient salt-doping in organic semiconductors†

**DOI:** 10.1039/d1tc06062g

**Published:** 2022-08-24

**Authors:** A. Rahimichatri, J. Liu, F. Jahani, L. Qiu, R. C. Chiechi, J. C. Hummelen, L. J. A. Koster

**Affiliations:** Zernike institute for Advanced Materials, University of Groningen, Nijenborgh 4 9747 AG Groningen The Netherlands l.j.a.koster@rug.nl; Stratingh Institute for Chemistry, University of Groningen, Nijenborgh 4 9747 AG Groningen The Netherlands

## Abstract

Doping to enhance the electrical conductivity of organic semiconductors is not without its challenges: The efficacy of this process depends on many factors and it is not always clear how to remedy poor doping. In the case of doping with salts, one of the possible causes of poor doping is a limited yield of integer charge transfer resulting in the presence of both cations and anions in the film. The charge of such ions can severely limit the electrical conductivity, but their presence is not easily determined. Here we introduce a set of simple conductivity measurements to determine whether poor doping in the case where the dopant is a salt is due to limited integer charge transfer. By tracking how the conductivity changes over time when applying a bias voltage for an extended amount of time we can pinpoint whether unwanted ions are present in the film. Firstly, we introduce the principle of this approach by performing numerical simulations that include the movement of ions. We show that the conductivity can increase or decrease depending on the type of ions present in the film. Next, we show that the movement of these dopant ions causes a build-up of space-charge, which makes the current–voltage characteristic non-linear. Next, we illustrate how this approach may be used in practice by doping a fullerene derivative with a series of organic salts. We thus provide a tool to make the optimization of doping more rational.

## Abstract 2

To assess if salt-doping leaves anions and cations in the film, limiting the conductivity, one can apply a bias voltage and monitor the conductivity over time. If the doping is limited by unwanted ions, then the conductivity will increase with time.

## Graphical abstract

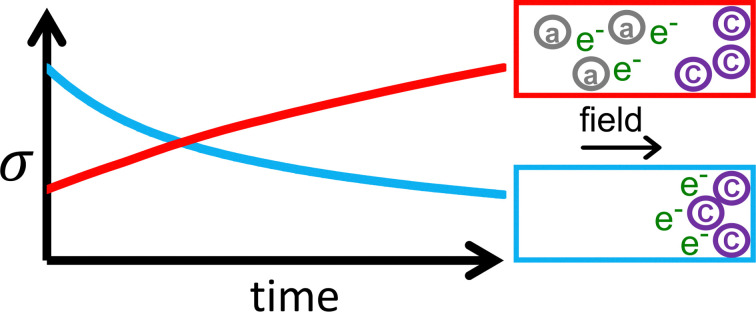

## 1 Introduction

Doping enhances the electrical conductivity of organic semiconductors (OSCs) by increasing the free charge carrier density and enables control of their electronic properties.^1–6^ N-type or p-type doping have been widely used to improve charge transport and selectivity of electrodes in many organic electronic devices, including organic solar cells, organic light emitting diodes, organic memristors, organic thermoelectrics and hybrid organic–inorganic perovskite solar cells.^1,4,6–14^ The development of n-type doping is still lagging because of the relatively low conductivities of n-doped solution-processed organic semiconductors compared to their p-type counterparts, caused by low doping efficiency.

Several factors are responsible for ineffective doping in OSCs. It is known that in efficiently doped OSCs, doping is the result of integer charge transfer between host and dopant molecules, followed by the generation of free charge carriers.^15^ However, if integer charge transfer has a low yield, then the doping will be less effective. In the case of doping with salts, integer charge transfer with a limited yield results in the presence of both cations and anions in the film: In an n-doped (p-doped) film, any remaining anions (cations) reduce the electrical conductivity. The presence of such unwanted ions is not easily detected and, thus, it is not clear what limits the conductivity without extensive additional experiments. Müller *et al.* have shown that dopant ions can drift upon the application of an electric field, thereby de-doping part of the film, leading to a loss in conductivity.^16^ The reversible drift of dopant ions was investigated using electrical measurements, optical microscopy, spatially resolved infra-red spectroscopy, and scanning Kelvin probe microscopy.^16^

In this work, we identify the cause of inefficient salt-doping by making use of the ability of dopant ions to drift. For example, in the case of efficient n-doping, integer charge transfer is quantitative and we have cations only. In contrast, if integer charge transfer has a yield less than unity, doping is inefficient and there will be cations and anions in the film. We show, by means of simulations, that these two cases can be discriminated by monitoring the electrical conductivity over time when applying a bias voltage. The current–time behaviour under the applied bias voltage is strongly influenced by the mobile ionic species: if the anions are more mobile than the cations the current rises, while if the cations are mobile the current decays.

In order to demonstrate this approach and how it can be applied to experimental data, we tested several doped devices using a fullerene derivative as the host molecule and four different organic salt n-dopants, namely, tetrabutylammonium fluoride (TBAF), tetrametylammonium fluoride (TMAF), tetrabutylammonium iodide (TBAI) and tetrabutylammonium tetrafluoroborate (TBABF_4_). We found that highly conductive devices show a current decrease which is a signature of a good doping, where dopant cations are mobile, with smaller cations leading to a faster decrease in the current. On the other hand, weakly doped devices studied herein show a current rise under a bias voltage, which is evidence of the anions being more mobile. Finally, we show that the temporal response of the current can be helpful to identify inefficient doping in doped OSC devices.

## 2 Numerical case study

Before we turn to the experiments, we use an open-source numerical drift-diffusion simulation program^17,18^ that solves the electronic and ionic contributions to the conductivity in order to simulate how the drift of dopant ions influences the conductivity behaviour for films of salt-doped OSCs. For the case of n-type doping, it is instructive to consider three idealized cases of n-type doping:

• Case I: quantitative charge transfer.

• Case II: limited yield of integer charge transfer, leading to cations and anions dispersed in the film. Anions are mobile.

• Case III: limited yield of integer charge transfer, leading to cations and anions dispersed in the film. Cations are mobile.

These cases are not based on any specific host-dopant system. Rather, they correspond to cases where the ions of the salt are dispersed in the film. Then, depending on Case I, II, and III, either all anions transfer an electron to the host (Case I) or only a fraction (Cases II and III) do so. All of this is assumed to happen upon the fabrication of the device, *i.e.* well before applying the bias voltage. Once the voltage is applied, any ions in the film can drift in the electric field, which changes the local doping level and, hence, electrical conductivity. In the simulations we assume, for the sake of simplicity, that there are no solubility issues in the doping process, in other words, all ionic species are assumed to disperse in the film. In the cases of limited integer charge transfer (II and III), we assume that half of the dopant anions transfer their charge to the host, while the other half remains negatively charged.

To simulate the conductivity behaviour of Cases I, II, and III, we simulate the current flowing in an n-doped organic layer. Initially, the voltage is rapidly swept up from 0 to 6 V (corresponding to an average electric field of 2 V μm^−1^). Next, the voltage is kept constant for considerable time and, lastly, the voltage is swept back to zero. Further details of the numerical simulations can be found in the ESI.†

**Fig. 1:**
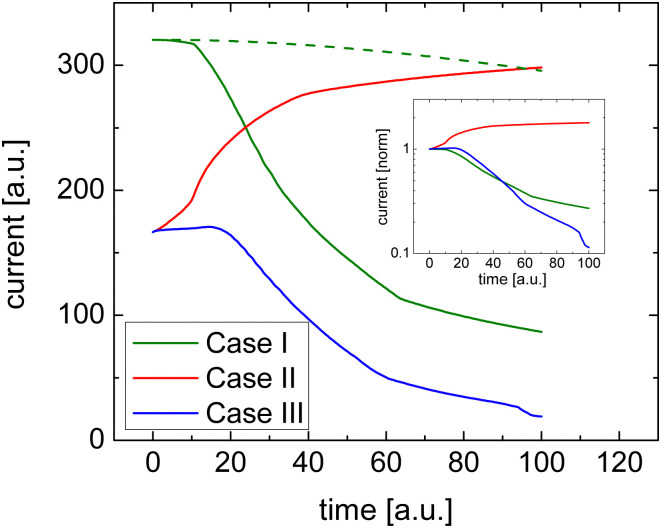
Numerical simulations of the change in current upon the application of a bias voltage stress for the three different cases of doping. The effect of a threefold reduction of the mobility of dopant ions is shown for Case I (dashed line). The inset shows the data normalized to the initial current.

Fig. 1 shows how efficient charge transfer (Case I) yields a highly conductive film and confirms that the current decreases over time consistent with the work by Müller *et al.*^16^ Initially, the conductivity is high due to the presence of salt cations. These ions compensate the space charge of the electrons. As a result, there are many electrons in the film and the current is high. As the space charge is compensated and the electric field is constant, the initial current–voltage characteristic of such a uniformly doped film is linear, which is also what is seen in typical experiments.

Upon the application of a bias voltage, the dopant ions start to drift and the conductivity decreases. The rate of the loss of conductivity is, unsurprisingly, related to the mobility of the dopant ions. As indicated by the dashed line in Fig. 1 the decay is slower if the mobility of dopant ions is reduced.

**Fig. 2:**
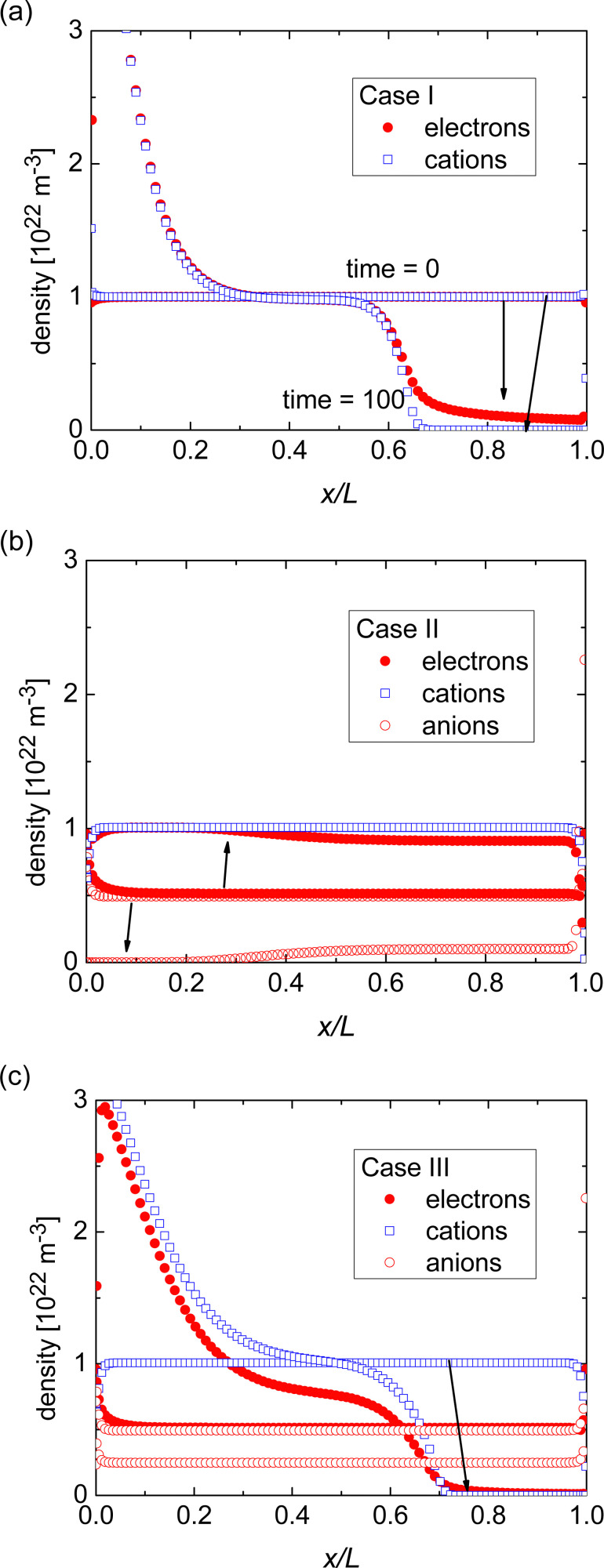
Simulated profiles of the electron, cation, and anion densities for Cases I, II, and III. The arrows indicate how the density profiles change from before to after the application of the bias voltage stress.

**Fig. 3:**
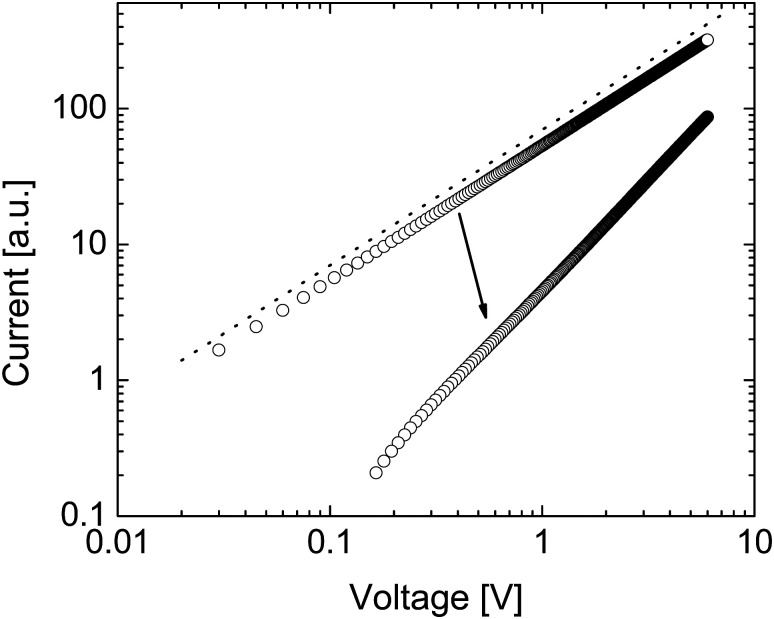
Simulated current–voltage characteristic corresponding to Case I before and after application of bias voltage (the arrow indicates the change in current upon stressing the system). Upon bias voltage stress, the current decreases and becomes super-linear (the dashed line has a slope of 1), due to a build-up of net space charge in the film.

Fig. 2(a) shows how the electron density and cation density profiles change within the doped film. Upon the application of the bias voltage, the dopant cations drift towards the injecting electrode, thereby de-doping the bulk of the film. As a result, the negative charge of the electrons in the bulk of the film is no longer compensated, leading to a decrease in electron density and concomitant loss of conductivity. As the bulk of the film is de-doped, the electric field across the film is no longer uniform (data not shown). A macroscopic, measurable consequence of this is a qualitatively different behaviour of the current upon a fast sweep of the bias voltage. Fig. 3 illustrates how a fast current–voltage sweep changes from completely linear (before the application of a long bias voltage) to a super-linear one after the application of a long bias voltage stress. This non-linearity is caused by the space-charge within the de-doped part of the layer. This space-charge distorts the electric field, not unlike space-charge-limited current.^19^ One can thus use a quick current–voltage scan after the application of a bias voltage stress to confirm that the loss of conductivity is not (only) due to a loss of dopants or unfavourable chemical changes to the host material.

The response in Cases II and III is less obvious, see Fig. 1. In either case, the initial conductivity is low.§§The simulations shown were performed on relatively narrow electrode spacing (3 μm) due to numerical constraints. Additionally, it is assumed that cations and anions are distributed throughout the entire film. In reality, it is likely that inefficient charge-transfer is accompanied by severe phase-separation of the host and dopant. Both assumptions likely result in conductivities that are relatively high as compared to the experimental situation. These cases, corresponding to integer charge transfer with a limited yield, can either show an increase or decrease of the conductivity over time, depending on which ionic species (cations or anions) is the more mobile one. If anions are more mobile than cations (Case II), then the mobile anions drift towards the electrodes, leaving behind the cations to effectively dope the bulk of the film. As a result, the electron density increases and so does the conductivity (see Fig. 2(b)). Case III, on the other hand, shows the opposite trend (see Fig. 2(c)): the more mobile cations drift to the electrode, and the bulk of the film becomes effectively p-doped due to presence of anions. The electron density, consequently decreases as does the conductivity.

To summarize the numerical simulations results, the initial conductivity is high if charge transfer is efficient (Case I), followed by a decreasing conductivity upon applying a bias voltage. One can verify that this decrease is not due degradation of the doped film but rather due to de-doping by checking the linearity of a fast current–voltage scan soon after the application of the bias voltage. Inefficient charge transfer (Cases II and III), however, shows up as a modest/low initial conductivity followed by either an increase or decrease of the current depending on the relative mobility of the ionic species.

## 3 Experimental results

Müller and co-workers demonstrated that dopant ion movement can occur at the moderate field strength of 1 V μm^−1^.^16^ In our study, we use a fixed electric field of 2 V μm^−1^ as this is enough to move the ions, but small enough to ensure that the charge carrier mobility is still in the regime where it is constant.

**Fig. 4:**
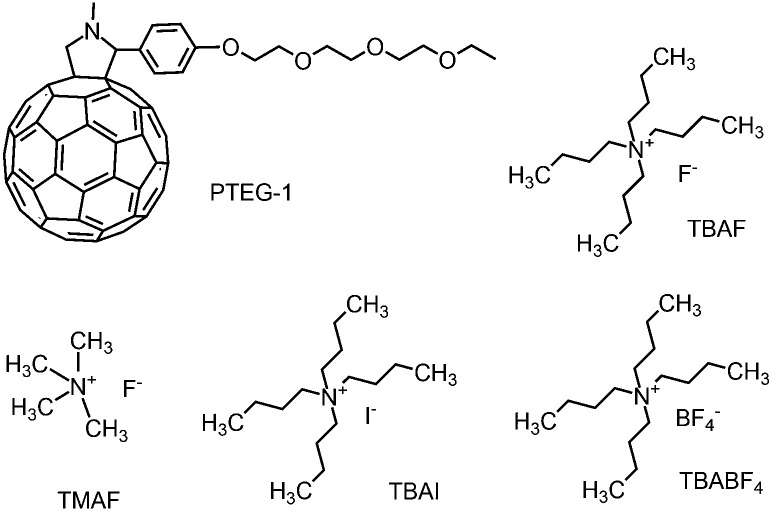
Chemical structures of the host and dopant molecules used in this work.

We used the fullerene derivative PTEG-1 (see Fig. 4)^6^ as the host molecule and tested several doped OSC devices using four different organic salt dopant molecules, containing alkyl ammonium cations and various anions. The dopants used are TBAF, TMAF, TBAI and TBABF_4_, the structure of which are shown in Fig. 4. Dopant molecule concentrations of all the devices were 20 mol%. PTEG-1 doped devices were fabricated on top of an n-doped silicon substrate with a 230 nm thermally deposited oxide layer and lithographically patterned interdigitated Au electrodes. The channel length, channel width and thickness of all the devices were 5 μm, 10 mm and 100 nm, respectively. Prior to the experiments, each device was annealed at 120 °C for an hour for enhancing the doping efficiency.

As shown in Table 1, TBAF and TMAF doped films show relatively high conductivities of 0.43 and 0.25 S cm^−1^ respectively. Considering the strong basicity of F^−^, the high conductivities of TBAF and TMAF doped devices support that they undergo efficient charge transfer, leaving behind the dopant counter-ions (cations), in accordance with simulation Case I.

**Table 1:** Conductivities of doped PTEG-1 molecules using various dopants. TBAF and TMAF represent good n-dopants, whereas TBABF_4_ and TBAI are weak n-dopants

Sample	Conductivity [S cm ^−1^]
TBAF	0.43
TMAF	0.25
TBABF_4_	0.003
TBAI	0.002

**Fig. 5:**
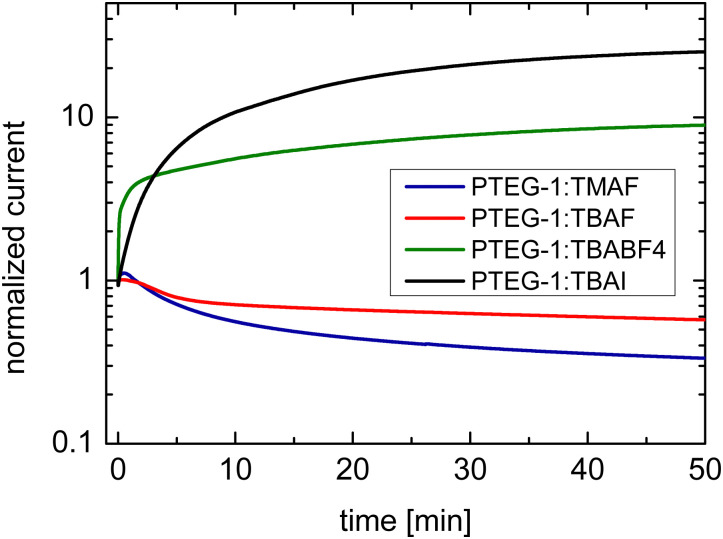
Temporal response of the current in TBAF, TMAF, TBAI, and TBABF_4_ doped devices under application of 2 V μm^−1^ bias electric field.

Upon the application of the long duration positive bias of 2 V μm^−1^ on TMAF and TBAF doped devices, the current decays, which is a result of the drift of the dopant cations under the electrical force of the bias and de-doping of the bulk of the film (Fig. 5 and Case I in Fig. 1), in accordance with the work of Müller *et al.*^16^ The decay is faster in the PTEG-1:TMAF film which has smaller cations. This suggests that the TMA^+^ cations move faster than TBA^+^ in the studied devices.

The drift of TMA^+^ and TBA^+^ cations upon application of the bias stress causes non-uniformity of the electric field across the device, which, as was predicted in Fig. 3, changes the current–voltage characteristic from linear (before the application of the bias voltage) to super-linear (after the application of the bias voltage) (see Fig. S2, ESI†). Therefore, the reduction of current with the bias voltage stress in TBAF and TMAF doped devices is not the result of unwanted chemical changes within the film or reducing the amount of the dopant ions in the film.

Negating the dopant ion movement under bias voltage can improve the long term performance of doped organic films under working conditions and enhance stability of the OSC devices. Reiser and co-workers have synthesized a new n-type dopant molecule which can create a covalent bond with a large variety of organic host molecules.^20^ The blend of this molecule, AzBnO-DMBI, with PCBM host was activated by UV light and generated highly reactive nitrenes to ultimately form covalent carbon–nitrogen bonds between PCBM and the dopant.^20^ Nevertheless, in most of the doped organic films with decent conductivities, dopant ions are not covalently bonded to the host which causes ionic drift or diffusion. In our study, although the presence of ethylene glycol (EG) side chain in PTEG-1 molecule enhances host/dopant compatibility and therefore provides a good doping effect,^21,22^ the size of EG side chain might influence the degrees of freedom of the ions within the host matrix and affect stability of the device, as it has been shown that the ionic dopant mainly resides in the plane of the polar side chains.^23^

The second set of doped devices, namely, PTEG-1:TBABF_4_ and PTEG-1:TBAI, have low conductivities of ∼0.003 S cm^−1^ and 0.002 S cm^−1^, respectively (Table 1), represent weak doping, possibly due to a limited yield of integer charge transfer. In this case, as mentioned in Section 2, application of a bias voltage may lead to either an increase or decrease of the current, which is determined by the faster moving ions.

TBABF_4_ and TBAI doped devices show a current rise upon application of the bias stress (Fig. 5), which is consistent with simulation Case II in Fig. 1 where the anions are more mobile (see Case II in Fig. 2). The drift of the anions towards the electrodes leads to further doping of the film and increasing of the conductivity. This comes as no surprise, as TBA^+^ is much bigger than either I^−^ and BF_4_^−^, with ionic radii of 4.94 Å, 2.06 Å, and 2.32 Å, respectively.^24–26^ The current rise is more pronounced in the TBAI device, which can be a projection of its slightly smaller anions.

Again, as expected, as a result of the electric field becoming non-uniform after the application of the bias voltage, the *I*–*V* sweep in Fig. S3 (ESI†) changes from linear to superlinear.

Subsequently, all the doped devices were left in inert atmosphere for 44 days. In Fig. S4 (ESI†), the *I*–*V* response of the doped devices after this period is shown. By removing the bias voltage and giving sufficient time, the ions appear to move back, as the linearity of the current–voltage curves of all four devices is restored. In the case of TMAF the conductivity is reduced by a factor of 10, showing that TMAF is less stable.

We attempted to estimate doping efficiencies or ion mobilities based on this work. However, this is complicated by the fact that the mobility of charge carriers in organic semiconductors is typically dependent on their density.^27^ So if the doping level changes as the experiment progresses, then the charge carrier mobility is also likely to change. Ultimately, the simulations serve to show that the current can also increase over time (Fig. 1), rather than only decrease. They also explain why this happens. After having established this, we can then reverse the argument: Some dopants work better than others. One of the possible causes is integer charge-transfer with a low yield, in the sense that not all dopants transfer a charge to the host and we have both positive and negative ions in the film. How can this be shown? Simply by applying a bias voltage and monitoring the current over time.

## 4 Conclusions

In conclusion, we have studied salt-doping of organic semiconductors in two scenarios: that of efficient integer charge-transfer—and resulting good conductivity—and that of integer charge-transfer with a limited yield, which leads to low conductivity. This prompted the introduction of a simple conductivity measurement protocol that allows us to identify the types of ions present and mobile in the films. Using this protocol, we have been able to attribute the low conductivity (TBAI and TBABF_4_ doping) to the limited yield of integer charge-transfer, rather than to other possible factors such as morphological issues.

Thus, the temporal response of the current under an applied bias voltage can be used as a means to identify one of the possible causes of inefficient doping. Furthermore, we can use the current–voltage curve as a means to check if the field in the film is still uniform after removal of the bias stress, which also holds for p-doped OSCs. The tool introduced in this work can be used to simplify the development of efficient doping strategies of organic semiconductors.

## 5 Experimental procedures

Materials: PTEG-1 was synthesized according to a previously reported procedure.^28^ TBAF, TMAF, TBAI, TBABF_4_ were purchased from Sigma Aldrich.

Device fabrication: 15 × 15 mm^2^ n-doped silicon substrates coated with 230 nm SiO_2_ and patterned with 30 nm Au interdigitated electrodes were used as substrate. The substrates were ultra-sonicated with acetone (3 times, each for 20 min) to remove the resist protection layer AZ7217, followed by iso-propanol ultra-sonication for 20 min. The substrates were then dried with nitrogen gun and transferred into the oven at 140 °C, followed by UV-ozone treatment for 20 min.

PTEG-1 (>99%) solutions (10 mg mL^−1^ in chloroform) were mixed with different amounts of dopant solution (5 mg mL^−1^ in chloroform/ethanol) in nitrogen atmosphere to get 20 mol% doped solutions. The solutions were spin-coated at 1000 rpm for 40 seconds, yielding films of ∼100 nm). The devices were annealed at 120 °C for one hour, prior to *I*–*V* measurements. Devices with channel width of 10 mm and channel length of 5 μm were used for all measurements.

Measurements: current–voltage and current–time measurements were conducted in a probe station under nitrogen atmosphere, using a Keithley 2000 source meter.

## Conflicts of interest

There are no conflicts to declare.

## Supplementary Material

TC-010-D1TC06062G-s001

## References

[cit1] Bin Z., Liu Z., Qiu Y., Duan L. (2018). Adv. Opt. Mater..

[cit2] Gregg B. A., Chen S.-G., Cormier R. A. (2004). Chem. Mater..

[cit3] Pfeiffer M., Leo K., Zhou X., Huang J., Hofmann M., Werner A., Blochwitz-Nimoth J. (2003). Org. Electron..

[cit4] Wang Z., McMeekin D. P., Sakai N., van Reenen S., Wojciechowski K., Patel J. B., Johnston M. B., Snaith H. J. (2017). Adv. Mater..

[cit5] Werner A. , Pfeiffer M., Harada K., Leo K. and Elliot C., N-Doping Of Organic Semiconductors, 2007, US Patent App. 10/595,319

[cit6] Liu J., Qiu L., Portale G., Koopmans M., Ten Brink G., Hummelen J. C., Koster L. J. A. (2017). Adv. Mater..

[cit7] Bin Z., Li J., Wang L., Duan L. (2016). Energy Environ. Sci..

[cit8] Kim J. H., Liang P.-W., Williams S. T., Cho N., Chueh C.-C., Glaz M. S., Ginger D. S., Jen A. K.-Y. (2015). Adv. Mater..

[cit9] Mahmood K., Sarwar S., Mehran M. T. (2017). RSC Adv..

[cit10] Stubhan T., Litzov I., Li N., Salinas M., Steidl M., Sauer G., Forberich K., Matt G. J., Halik M., Brabec C. J. (2013). J. Mater. Chem. A.

[cit11] Xia F., Wu Q., Zhou P., Li Y., Chen X., Liu Q., Zhu J., Dai S., Lu Y., Yang S. (2015). ACS Appl. Mater. Interfaces.

[cit12] Hu L., Liu T., Duan J., Ma X., Ge C., Jiang Y., Qin F., Xiong S., Jiang F., Hu B. (2017). et al.. Adv. Funct. Mater..

[cit13] Yang Q., Hao Y., Wang Z., Li Y., Wang H., Xu B. (2012). Synth. Met..

[cit14] Drechsel J., Männig B., Kozlowski F., Pfeiffer M., Leo K., Hoppe H. (2005). Appl. Phys. Lett..

[cit15] Schwarze M., Gaul C., Scholz R., Bussolotti F., Hofacker A., Schellhammer K. S., Nell B., Naab B. D., Bao Z., Spoltore D., Vandewal K., Widmer J., Kera S., Ueno N., Ortmann F., Leo K. (2019). Nat. Mater..

[cit16] Müller L., Rhim S.-Y., Sivanesan V., Wang D., Hietzschold S., Reiser P., Mankel E., Beck S., Barlow S., Marder S. R., Pucci A., Kowalsky W., Lovrincic R. (2017). Adv. Mater..

[cit17] Koopmans M., Le Corre V. M., Koster L. J. A. (2022). J. Open Source Softw..

[cit18] SIMsalabim, 2021, https://github.com/kostergroup/SIMsalabim

[cit19] Lampert M. and Mark P., Current Injection in Solids, Academic Press; New York, 1970

[cit20] Reiser P., Benneckendorf F. S., Barf M. M., Müller L., Bäuerle R., Hillebrandt S., Beck S., Lovrincic R., Mankel E., Freudenberg J., Jänsch D., Kowalsky W., Pucci A., Bunz U. H., Müllen K. (2019). Chem. Mater..

[cit21] Qiu L., Liu J., Alessandri R., Qiu X., Koopmans M., Havenith R. W., Marrink S. J., Chiechi R. C., Koster L. J. A., Hummelen J. C. (2017). J. Mater. Chem. A.

[cit22] Liu J., Qiu L., Portale G., Torabi S., Stuart M. C., Qiu X., Koopmans M., Chiechi R. C., Hummelen J. C., Koster L. J. A. (2018). Nano Energy.

[cit23] Liu J., Garman M. P., Dong J., van der Zee B., Qiu L., Portale G., Hummelen J. C., Koster L. J. A. (2019). ACS Appl. Energy Mater..

[cit24] Poli I., Eslava S., Cameron P. (2017). J. Mater. Chem. A.

[cit25] Bureau H., Keppler H., Métrich N. (2000). Earth Planet. Sci. Lett..

[cit26] Xu K. , Ding S. and Jow T., Nonaqueous Electrolyte Development for Electrochemical Capacitors, US army research laboratory technical report, 1999

[cit27] Pasveer W. F., Cottaar J., Tanase C., Coehoorn R., Bobbert P. A., Blom P. W. M., de Leeuw D. M., Michels M. A. J. (2005). Phys. Rev. Lett..

[cit28] Jahani F., Torabi S., Chiechi R. C., Koster L. J. A., Hummelen J. C. (2014). Chem. Commun..

