# Supplementary material for: A method for identifying the cause of inefficient salt-doping in organic semiconductors
Source: J Mater Chem C Mater. 2022 Aug 24;10(36):13093–8. doi: 10.1039/d1tc06062g (PMC9494613; doi:10.1039/d1tc06062g)
Supplement: TC-010-D1TC06062G-s001 [file TC-010-D1TC06062G-s001.pdf]

Electronic Supplementary Material (ESI) for Journal of Materials Chemistry C.
This journal is © The Royal Society of Chemistry 2022

Electronic Supporting Information

—

A method for identifying the cause of inefficient salt-doping in organic semiconductors

A. Rahimi Chatri,[a] J. Liu,[a] F. Jahani,[a,b] L. Qiu,[a,b,c] R. C. Chiechi,[a,b] J. C. Hummelen[a,b] and L. J. A. Koster*[a]

[a] *Zernike institute for Advanced Materials, University of Groningen, Nijenborgh 4, 9747 AG Groningen, The Netherlands.*

[b] *Stratingh Institute for Chemistry, University of Groningen, Nijenborgh 4, 9747 AG Groningen, The Netherlands.*

[c] *Present address: Yunnan Key Laboratory for Micro/Nano Materials & Technology, National Center for International Research on Photoelectric and Energy Materials, School of Materials and Energy, Yunnan University, Kunming 650091, P. R. China.*

*Email: l.j.a.koster@rug.nl

List of Figures

S1 Voltage sweep used in the simulations. 4

S2 Current- voltage characteristic of PTEG-1, n-doped with TBAF and TMAF, before (solid lines) and after (dotted lines) application of long duration bias stress. The voltage sweep rate is $4\,\mathrm{Vs^{-1}}$. 4

S3 Current-voltage characteristic of PTEG-1, n-doped with TBAI and $TBABF_4$, a) before, and b) after the application of a long duration bias voltage. The voltage sweep rate is $4\,\mathrm{Vs^{-1}}$. 5

S4 Current-voltage characteristics of the n-doped devices, 44 days after application of long duration bias voltage, in logarithmic and linear scale. 6

1 Numerical procedures

We use a transient drift-diffusion program to simulate the time-voltage-current characteristics of a doped layer. The simulation code is open-source[(1)] and is available on GitHub.[(2)] The main simulation parameters are listed in Table S1. Figure S1 shows how the voltage is varied over time. The voltage is ramped up from 0 V to 6 V in 1 ms and held there for 100 s followed by a decrease back to 0 V in 1ms.

The simulation solves the transient development of the potential $V(x)$, electron density $n(x)$, and the ionic densities $n_{\text{ion}}(x)$ and $p_{\text{ion}}(x)$. The Poisson equation relates the potential $V(x)$ to the charge density

$$\frac{\partial}{\partial x}\left(\varepsilon\frac{\partial V(x)}{\partial x}\right) = q(n(x) + n_{\text{ion}}(x) - p_{\text{ion}}(x)). \tag{1}$$

where ε is the dielectric constant.

The ionic and electron current densities are solved from the drift-diffusion equations, then one has for electrons

$$J_n(x) = -qn(x)\mu_n(x)\frac{\partial V(x)}{\partial x} + kT\mu_n(x)\frac{\partial n(x)}{\partial x}, \tag{2}$$

and for positive ions, one has

$$J_{p\text{ion}}(x) = -qp_{\text{ion}}(x)\mu_{p\text{ion}}(x)\frac{\partial V(x)}{\partial x} - kT\mu_{p\text{ion}}(x)\frac{\partial p_{\text{ion}}(x)}{\partial x}, \tag{3}$$

where $\mu_{p\text{ion}}$ is the ion mobility. For negative ions, we have

$$J_{n\text{ion}}(x) = -qn_{\text{ion}}(x)\mu_{n\text{ion}}(x)\frac{\partial V(x)}{\partial x} + kT\mu_{n\text{ion}}(x)\frac{\partial n_{\text{ion}}(x)}{\partial x}. \tag{4}$$

The total current density is then given by

$$J(x) = J_n(x) + J_D(x) + J_{n\text{ion}}(x) + J_{p\text{ion}}(x), \tag{5}$$

where $J_D(x)$ is the displacement current.

The boundary condition on the potential V is given by

$$V(L) - V(0) = V_a, \tag{6}$$

where L is the thickness of the layer and V_a is the applied voltage. For the electron densities, we have the requirement that

$$n(0) = n(L) = N_c \exp(-\phi/kT), \tag{7}$$

where N_c is the effective density of states and ϕ is the injection barrier. The boundary condition on the ionic densities is set by requiring that no ions can enter or leave the layer through the electrodes.

Table S1: Parameters used in the transient drift-diffusion simulations.

parameter	value
device thickness	3000 nm
relative dielectric constant	4
effective density-of-states	2.5×10^{25} m^{-3}
electron mobility	10^{-7} m^2/Vs
injection barrier	0.2 eV
ion concentration	10^{22} m^{-3}
ion mobility (high)	10^{-13} m^2/Vs
ion mobility (low)	10^{-18} m^2/Vs

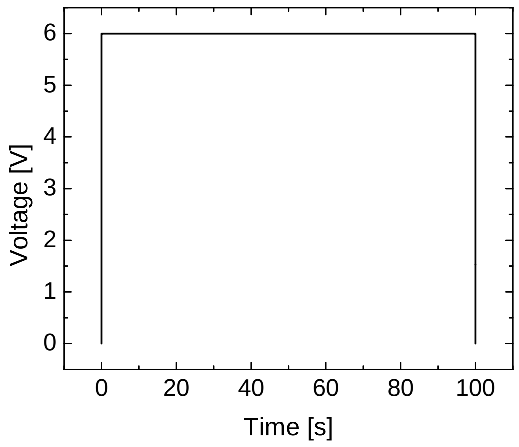

Figure S1: Voltage sweep used in the simulations.

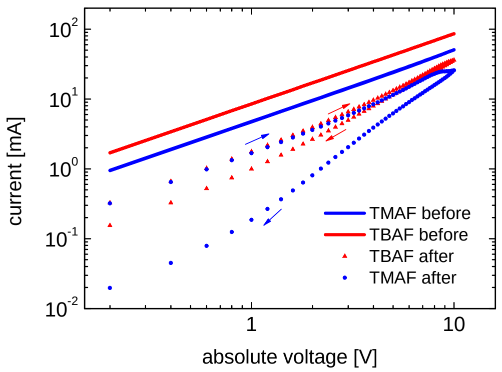

Figure S2: Current- voltage characteristic of PTEG-1, n-doped with TBAF and TMAF, before (solid lines) and after (dotted lines) application of long duration bias stress. The voltage sweep rate is 4 Vs^{-1}.

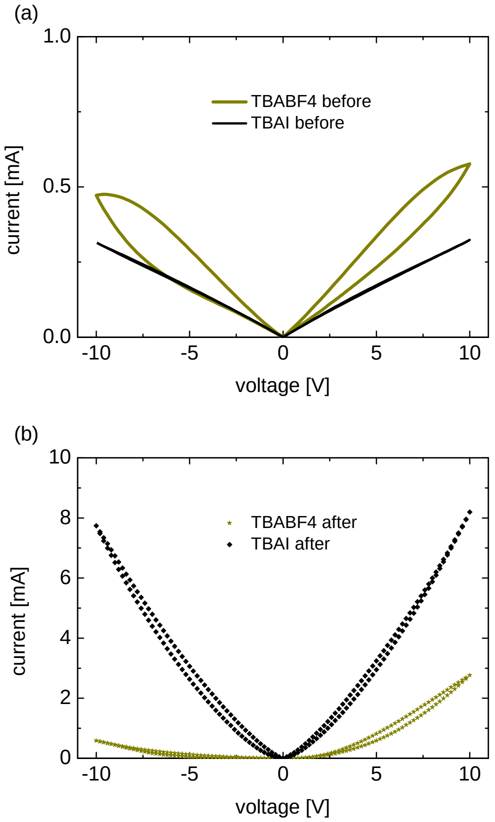

Figure S3: Current-voltage characteristic of PTEG-1, n-doped with TBAI and $TBABF_4$, a) before, and b) after the application of a long duration bias voltage. The voltage sweep rate is $4\,Vs^{-1}$.

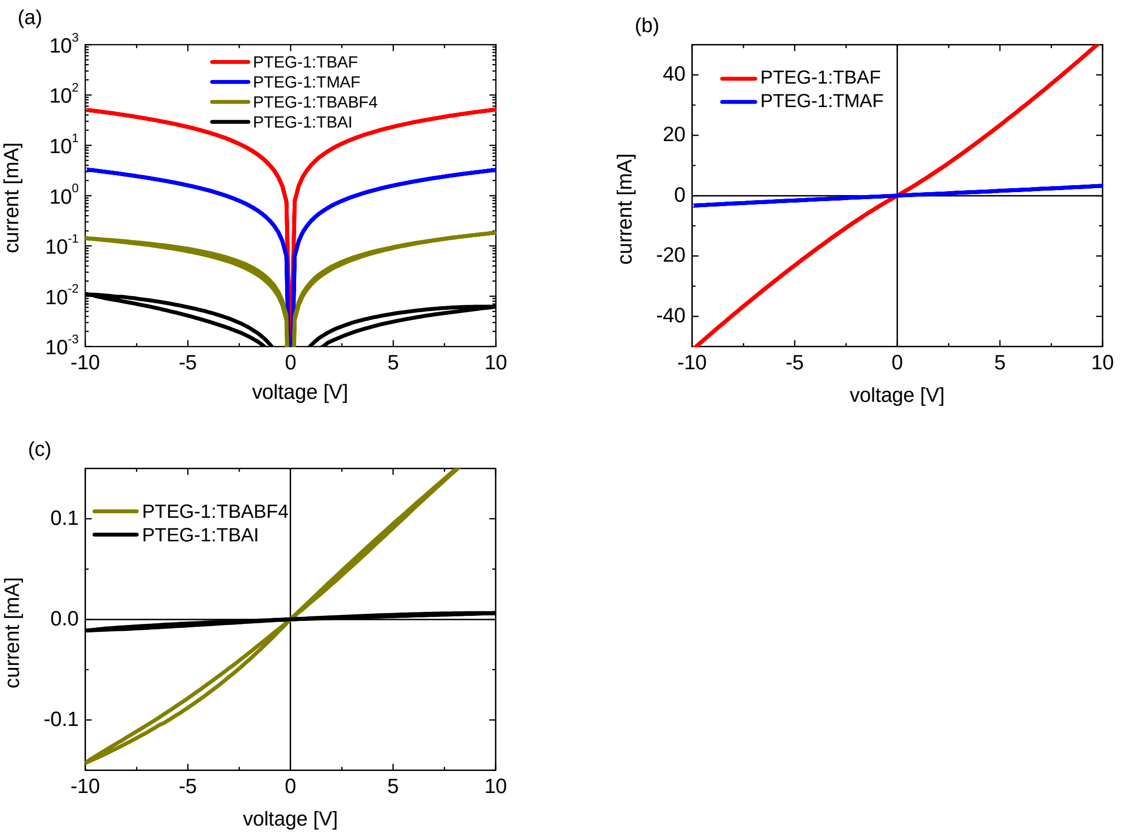

Figure S4: Current-voltage characteristics of the n-doped devices, 44 days after application of long duration bias voltage, in logarithmic and linear scale.

2 Experimental results

References

[1] M. Koopmans, V. M. Le Corre and L. J. A. Koster, *J. Open Source Softw.*, 2022, **7**, 3727.

[2] *SIMsalabim*, 2021, https://github.com/kostergroup/SIMsalabim.
